# Holographic Quantitative Structure-Activity Relationships of Tryptamine Derivatives at NMDA, 5HT_1A_ and 5HT_2A_ Receptors

**DOI:** 10.3390/molecules18088799

**Published:** 2013-07-24

**Authors:** Rungtiva Palangsuntikul, Heinz Berner, Michael L. Berger, Peter Wolschann

**Affiliations:** 1 Biological Engineering Program, Faculty of Enigineering, King Mongkut’s University of Technology Thonburi, Bangmod campus, Bangkok 10140, Thailand; 2 Institute for Theoretical Chemistry, University of Vienna, Währinger Strasse 17, Vienna 1090, Austria; 3 Center for Brain Research, Medical University of Vienna, Spitalgasse 4, Vienna 1090, Austria

**Keywords:** HQSAR, NMDA receptor, spermine, tryptamine, 5HT_2A_ receptor, 5HT_1A_ receptor

## Abstract

Tryptamine derivatives (Ts) were found to inhibit the binding of [^3^H]MK-801, [^3^H]ketanserin and [^3^H]8-OH-DPAT to rat brain membranes. [^3^H]MK-801 labels the NMDA (N-methyl-D-aspartate) receptor, a ionotropic glutamate receptor which controls synaptic plasticity and memory function in the brain, whereas [^3^H]ketanserin and [^3^H]8-OH-DPAT label 5HT_2A_ and 5HT_1A_ receptors, respectively. The inhibitory potencies of 64 Ts (as given by IC_50_ values) were correlated with their structural properties by using the Holographic QSAR procedure (HQSAR). This method uses structural fragments and connectivities as descriptors which were encoded in a hologram thus avoiding the usual problems with conformation and alignment of the structures. Four correlation equations with high predictive ability and appropriate statistical test values could be established. The results are visualized by generation of maps reflecting the contribution of individual structural parts to the biological activities.

## 1. Introduction

Ionotropic glutamate receptors are responsible for glutamate-mediated excitatory transmission in mammalian brain [1,2]. 5HT_2A_ and 5HT_1A_ receptors are probably the most prominent subtypes of the exuberant family of serotonin receptors. Several modeling studies have dealt with these two targets [3,4,5,6,7,8,9,10], most of them based on SAR considerations by using solely synthetic drugs as ligands. Until now structural variants of the natural ligand 5-OH-tryptamine were not investigated in great detail. In this work, potencies of tryptamine derivatives (Ts) as inhibitors of [^3^H]MK-801 [11] binding (in the absence or presence of a stimulating spermine concentration) and as inhibitors of [^3^H]ketanserin [12], and [^3^H]8-OH-DPAT [13] binding to rat brain membranes were used to generate and evaluate holographic quantitative structure activity relationship (HQSAR) models.

The fragment-based HQSAR turned out to be a promising tool for establishing a relationship between the structure of a compound and its biological activities. It is a modern QSAR technique that requires no explicit 3D information for the ligands (e.g., structure, physicochemical descriptors, conformation and molecular alignment) [14]. As it is done with 3D-QSAR techniques, such as CoMFA and CoMSIA, HQSAR as well could easily and rapidly generate QSAR models with high predictive value for both small and large data sets [15].

## 2. Results and Discussion

Basically, this analysis involves three main steps: (1) generation of structural fragments for each T; (2) the encoding of these fragments into a molecular hologram; (3) the statistical generation of PLS QSAR models [16]. In our studies, the influence of the fragment distinction parameters on the statistical values of our models was investigated. Thus, several combinations of fragment parameters were considered during the QSAR modeling runs.

### 2.1. HQSAR of Ts as Inhibitors of [^3^H]MK-801 Binding

A set of 64 Ts were used to establish a HQSAR model for the inhibition of [^3^H]MK-801 binding. The optimum number of components was selected for each fragment parameter combination. We have calculated 63 parameter combinations. Only a set of six combinations is given in Table 1 (the others show less significant results). 

**Figure 1:**
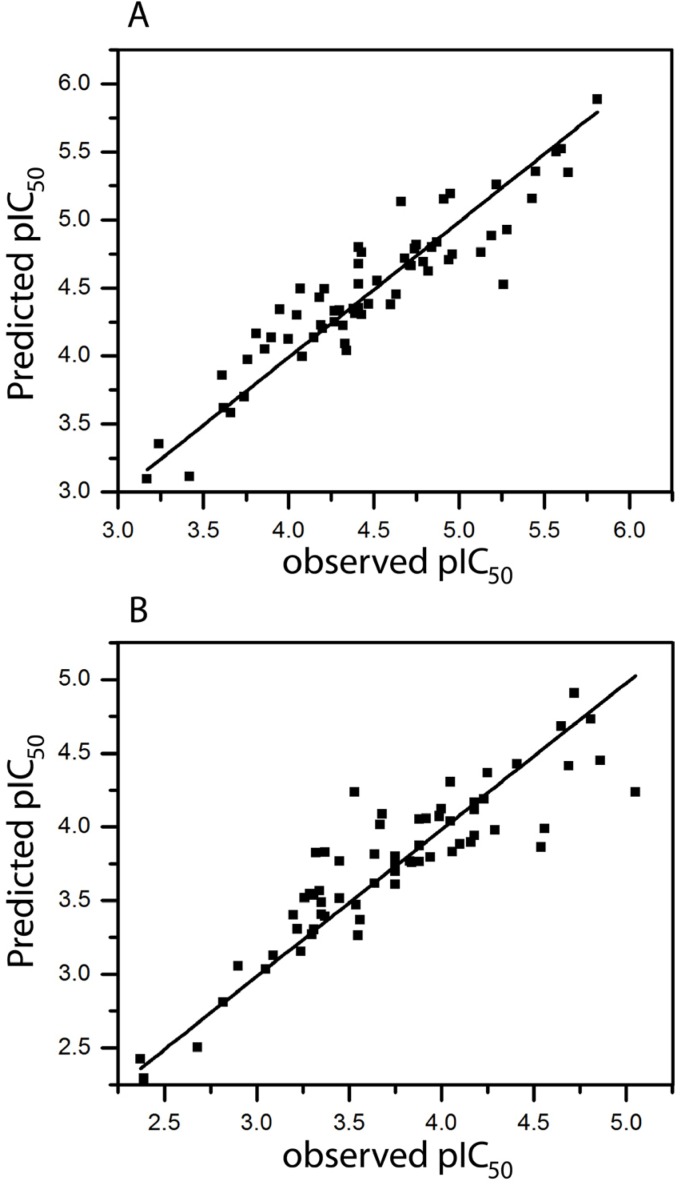
Plot of predicted *versus* observed pIC_50_ value of Ts inhibiting (**A**) [^3^H]MK-801 binding (model 63, Table 1) and (**B**) [^3^H]MK-801 binding in presence of 30 µM spermine (model 63, Table 2).

The donor & acceptor parameter (model 6) seems to play a certain role for inhibitory activity, while hydrogen atoms (model 4) and chirality (model 5) show a small q^2^. By adding more fragment parameters to the model, continuously further improvements were observed (models 15, 28, 50, 58, 63). Model 63, based on a combination of all fragments gave the best result (q^2^ = 0.632 and r^2^ = 0.855). This model was derived using six fragment distinctions, with six being the optimum number of PLS components. The plot of pIC_50_ values of all molecules predicted by model 63 *versus* experimental values is shown in Figure 1A. As can be seen, the predicted values are in good agreement with experimental values thus entailing a model with good correlative and predictive abilities.

The HQSAR-based fragmentation of a molecule into atoms allows to evaluate which of them are correlated with the biological activity of the molecule. HQSAR models can be graphically represented in the form of contribution maps where the color of each molecular fragment reflects the contribution of an atom or a small number of atoms to the activity of the molecule under study. The colors at the red end of the spectrum (*i.e.*, red, red-orange, and orange) reflect unfavorable (negative) contributions, while colors at the blue end (*i.e.*, yellow, green-blue and blue) indicate favorable (positive) contributions. Atoms with intermediate contributions are colored in white. The common backbones are colored cyan.

**Table 1:** Regression summary of HQSAR models of various combinations of fragment distinction parameters for 64 Ts as inhibitors of [^3^H]MK-801 binding. A, atoms; B, bonds; C, connections; H, hydrogen atoms; CH, chirality; DA, donor & acceptor; q^2^, LOO cross validated correlation coefficient; r^2^, non-cross-validated correlation coefficient; SE, standard error of estimate; ensemble: average value of ensemble q^2^ *; BL, best hologram length; n, number of components used in the PLS analysis.

Model	Field	n	q^2^	r^2^	SE	Ensemble	BL
6	DA	3	0.304	0.487	0.437	0.257	307
15	B/DA	3	0.451	0.591	0.390	0.365	59
28	A/C/DA	6	0.475	0.856	0.238	0.375	353
50	A/C/CH/DA	5	0.460	0.820	0.263	0.339	353
58	A/B/C/H/DA	6	0.539	0.832	0.256	0.398	61
63	A/B/C/H/CH/DA	6	0.632	0.855	0.238	0.399	61

* For each hologram length, a model could be established. The collection of these models comprises the ensemble.

**Figure 2:**
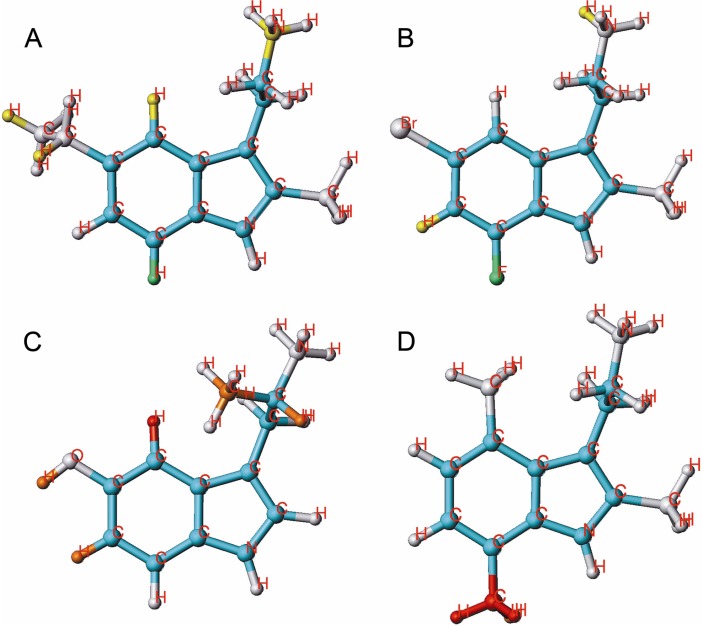
HQSAR contribution of molecular fragments to the inhibition of [^3^H]MK-801 binding; 2 examples for potent compounds **20** (**A**) and **58** (**B**), with favorable substituents in yellow and very favorable ones in green and 2 examples for weak compounds **40** (**C**) and **43** (D), with unfavorable substituents in orange and very unfavorable ones in red are given.

A red color code at position 5 was returned for compounds without substituent at this position: thus, there is an obvious demand for an appropriate substitution at this particular carbon (compound **1**). Methyl groups at positions 6, 7, α or N, respectively, are strongly disfavored. Several 2-Me-Ts with different alkyl substituents at position 5 (compounds **19**–**22**) contribute favorably to activity as indicated by the green and yellow color (compound **20** being the most potent, Figure 2A). Halogen substituents as well, especially fluorine (compound **47**), are positively contributing to the activity. On the other hand, hydroxy- or methoxy-substituents exert a weak to strong negative contribution. For instance, compound **40**, the least active compound of the series, carries a hydroxyl group at position 5 and a methyl group at position α, a highly unfavorable substituent pattern (Figure 2C).

Alkyl substituents at position 7 are exerting a negative effect whereas halogens at this position are tolerated. Halogen substituents at positions 5 and 7 (compound **58**, Figure 2B) are positively contributing to activity. These results imply that electronegative substituents at position 5 and 7 enhance activity. Compound **43**, being one of the least active compounds, carries methyl groups at positions 2, 4 and 7. The decisive negative influence comes from the methyl group at position 7 (Figure 2D).

### 2.2. HQSAR of [^3^H]MK-801 Binding in the Presence of Spermine

As reported previously [17] the polyamine spermine reduces the potency of most Ts as inhibitors of [^3^H]MK-801 binding. This effect may involve the recently described GluN1/GluN2B interface mediating polyamine stimulation of the NMDA receptor [18]. We have calculated 63 parameter combinations. Only a set of 6 combinations is given in Table 2 (the others show less significant results). 

In an effort to obtain the best model, combinations of different fragments were considered. The parameter hydrogen atoms (model 4) and chirality (model 5) correlate badly with the activity. On the other hand, connections (model 3) play a certain role. Furthermore, model 63, based on combination of all fragments gave the best result (q^2^ = 0.600 and r^2^ = 0.803). This result does not surprise as the above studies have shown that a combination of all fragment parameters give the best model for [^3^H]MK-801 binding. The plot of pIC_50_ values of all molecules predicted by model-63 *versus* experimental values are reported in Figure 1B.

**Table 2:** Regression summary of HQSAR models combined with various fragment distinction parameters for the 64 Ts as inhibitors of [^3^H]MK-801 binding in the presence of 30 µM of spermine ^a^.

Model	Field	n	q^2^	r^2^	SE	Ensemble	BL
3	C	6	0.292	0.640	0.366	0.207	61
9	A/H	3	0.356	0.535	0.405	0.292	353
28	A/C/DA	6	0.453	0.831	0.250	0.326	353
51	A/H/CH/DA	3	0.425	0.587	0.382	0.362	401
58	A/B/C/H/DA	6	0.525	0.791	0.278	0.357	61
63	A/B/C/H/CH/DA	6	0.600	0.803	0.270	0.366	61

^a^ Abbreviations as in Table 1.

The fragment contribution pattern in the presence of spermine is similar to that in its absence. The same model 63 proved as the best one. While it was remarkable that in the presence of spermine substituents in position 5 were less advantageous than without spermine (especially in the case of substituents larger than methyl, see compounds **14**, **16**, **20**, **22**, **23**, **25**, **29**), all these attenuated IC_50_ values were above 10 µM, more than 2/3 even above 100 µM. Therefore, it may not be justified to elaborate on these results in any more detail.

### 2.3. HQSAR of Ts as Inhibitors of [^3^H]ketanserin Binding

We have calculated 63 parameter combinations for the 64 Ts as inhibitors of [^3^H]ketanserin binding. Only a set of seven combinations is given in Table 3 (the others show less significant results). 

**Figure 3:**
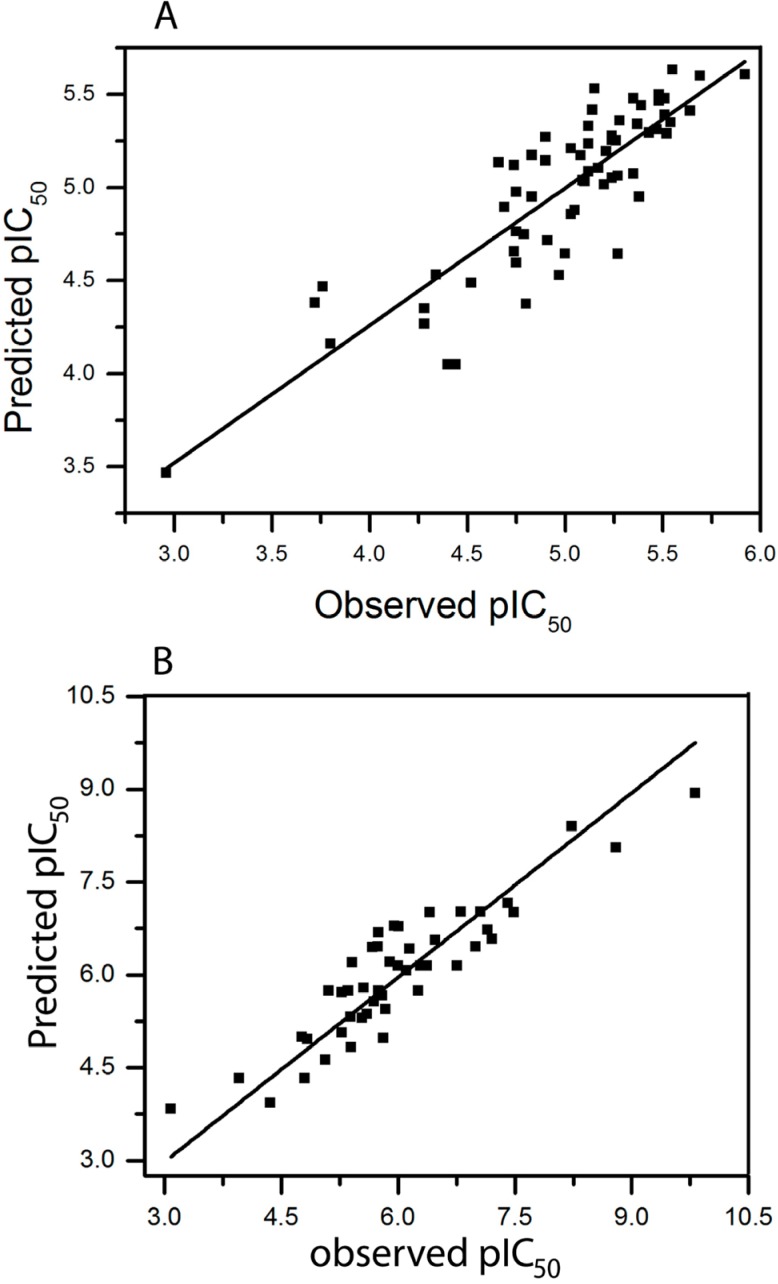
Plot of predicted *versus* observed pIC_50_ value of Ts inhibiting the binding of (**A**) [^3^H]ketanserin (model 58, Table 3); and (**B**) [^3^H]8-OH-DPAT (model 15, Table 4).

Model 3 (“connections”) yielded the most significant result. Adding hydrogen atoms (model 16) or chirality (model 17) did not improve the model. Also, the best combination of three descriptors, atoms, hydrogen atoms and donor & acceptor (A/H/DA, model 30) achieved only a small improvement. The best statistical result was obtained by model 58 which combines five descriptors (q^2^ = 0.489 and r^2^ = 0.738). The plot of pIC_50_ values of 59 Ts derivatives predicted by model 58-5 *versus* experimental values is reported in Figure 3A.

**Table 3:** Regression summary of HQSAR models combined with various fragment distinction parameters for the 64 Ts as inhibitors of [^3^H]ketanserin binding. ^a^

Model	Field	n	q^2^	r^2^	SE	Ensemble	BL
3	C	6	0.423	0.779	0.258	0.355	199
16	C/H	6	0.423	0.779	0.258	0.355	199
17	C/CH	6	0.423	0.779	0.258	0.355	199
30	A/H/DA	5	0.456	0.768	0.262	0.313	97
56	C/H/CH/DA	6	0.455	0.842	0.219	0.334	61
58	A/B/C/H/DA	3	0.489	0.738	0.274	0.341	199
63	A/B/C/H/CH/DA	5	0.465	0.794	0.247	0.328	199

^a^ Abbreviations as in Table 1.

**Figure 4:**
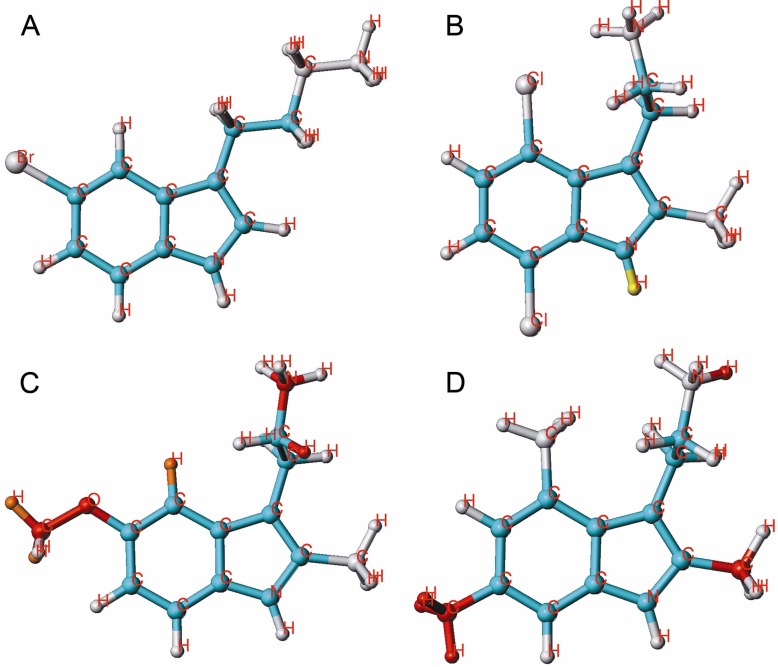
HQSAR contribution of molecular fragments to the inhibition of [^3^H]ketanserin binding; 2 examples for highly active compounds **14** (**A**) and **53** (**B**) and 2 examples for weak compounds, **29** (**C**) and **42** (**D**) are given.

A fragment contribution pattern including substituents in positions 1, 4 or 5 was favorable for high activity. A methyl substitution at the positions 6 and 7 was less advantageous, resulting in red and orange color coding (e.g., compound **42**, Figure 4D). However, combined substitution at positions 4, 5, 6 or 7 with methyl and halogen is tolerated. Substituents like CN, OH, OMe and CONH_2_ at position 5 are strongly disfavored (compound **29**, Figure 4C), while a halogen substituent at this position (*i.e*., compound **14**, Figure 4A) moderately contributed to activity. As we mentioned previously, also in this case the lack of coloring in A (Figure 4) it due to a slight advantage compared to compound **2** (orange in position 2 and 7). Combined with a 2-methyl substituent, halogens at positions 4, 5, 6 and 7 improve the inhibitory activity. Moreover, combined with 2-Me-substituted Ts alkyl groups can be at positioned at carbon 5 but not at carbon 7. The fragment contributions to the activity of molecule **53** (the most potent at this target) is displayed in Figure 4B.

### 2.4. HQSAR of Ts as Inhibitors of [^3^H]8-OH-DPAT Binding

We have calculated 63 parameter combinations for 47 Ts as inhibitors of [^3^H]8-OH-DPAT binding. Only a set of six combinations is given in Table 4 (the others show less significant results).

The fraction parameter connections (model 3) is very important, while a combination of the parameters bonds and donor & acceptor (model 15) is still improving the correlation. Using five (model 61) or all parameters (model 63) resulted in lower correlations. The best statistical result among all models was obtained for model 15 (q^2^ = 0.626 and r^2^ = 0.822). The plot of pIC_50_ values of all molecules predicted by model15 *versus* experimental values is shown in Figure 3B. 

**Table 4:** Regression summary of HQSAR models combined with various fragment distinction parameters for 47 Ts as inhibitors of [^3^H]8-OH-DPAT binding. ^a^

Model	Field	n	q^2^	r^2^	SE	ensemble	BL
3	C	4	0.575	0.753	0.610	0.521	83
15	B/DA	4	0.626	0.822	0.519	0.532	257
36	B/H/DA	4	0.626	0.822	0.519	0.532	257
55	B/H/CH/DA	4	0.626	0.821	0.519	0.533	257
61	A/C/H/CH/DA	5	0.574	0.830	0.512	0.436	71
63	A/B/C/H/CH/DA	4	0.530	0.792	0.560	0.445	97

^a^ Abbreviations as in Table 1.

**Figure 5:**
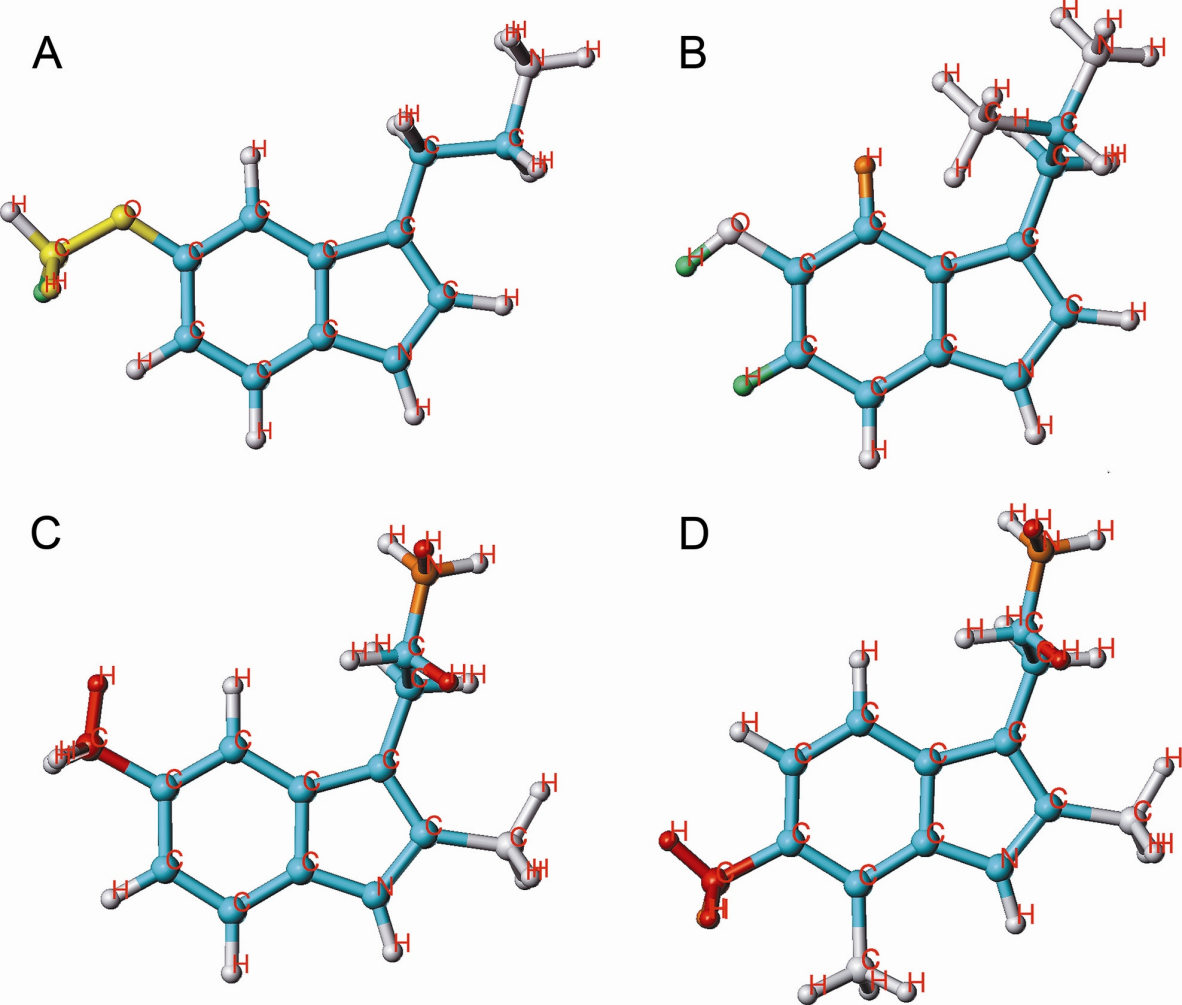
HQSAR contribution of molecular fragments to the inhibition of [^3^H]8-OH-DPAT binding; 2 examples for potent compounds **16** (**A**) and **40** (**B**) and 2 examples for weak compounds **19** (**C**) and **45** (**D**) are given.

Mono-alkylation (each position possible) has not much influence on activity. Two- and three-fold methyl-substituted derivatives, however (compound **19** and **45**, Figure 5C,D) exhibit reduced activity.

Individual atomic contributions of OH, OMe and CONH_2_ substituents at position 5 (most active compounds **15**, **16** (Figure 5A), **17** and **40** (Figure 5 B) are strongly associated with the biological activity of these compounds. The green and yellow colored pattern indicates their favorable contribution to the activity. Combined with a 2-methyl substitution, positions 5, 6 and 7 containing alkyl groups are strongly disfavoring activity, as it is shown by orange and red colored patterns. The modification of positions 4, 5, 6 and 7 with halogen substituents should be especially focused in order to improve the inhibitory activities of Ts.

## 3. Experimental

### 3.1. Data Set

Several Ts inhibit at micromolar concentration the four binding sites (5-HT_2A_ site, 5-HT_1A_ site, NMDA channel and polyamine-modulated NMDA channel). The affinities of 64 Ts (from sources as described [19]) at the NMDA channel were obtained from the inhibition of specific binding of the radioligand [^3^H]MK-801 (5 nM) to membranes prepared from hippocampal and parietal cortex of male Wistar rats. As reported previously [17], the potency of most Ts as inhibitors of [^3^H]MK-801 binding was reduced by the polyamine spermine (10 mM Tris acetate buffer pH 7.0, 30 µM spermine). The affinities at 5-HT_2A_ receptors were obtained from the inhibition of specific binding of [^3^H]ketanserin (1 nM) to rat prefrontal cortex membranes. In addition, 47 Ts were tested at 5-HT_1A_ receptors, as inhibitors of [^3^H]8-OH-DPAT binding (1 nM) to rat hippocampal membranes. Chemical structures together with experimental inhibition potencies are listed in Table A (see appendix).

At the chosen neutral pH, all Ts were in the protonated form. Concentrations of Ts providing 50% inhibition (IC_50_ values) were transferred to the corresponding pIC_50_ (–log IC_50_) values and used as dependent variables in the HQSAR analysis. In our previous work [19], we published an extended description of the data set together with the discussion of detailed pharmacological investigations.

### 3.2. HQSAR Analysis

The HQSAR modeling analyses, calculations and visualizations were performed using the SYBYL-X 1.0 package (Tripos Inc., St. Louis, MO, USA). HQSAR is a technique which employs fragment fingerprints as predictive variables of biological activity. These molecular fingerprints are broken into strings at fixed intervals as specified by the hologram length. The generation of the molecular fragments was carried out using several combinations of the following fragment distinction parameters: atoms (A), bonds (B), connections (C), hydrogen atoms (H), chirality (CH), and donor & acceptor (DA). Several combinations of these parameters were considered using fragment size of default 4–7. Each unique fragment in the data set is assigned a large positive integer by means of a cyclic redundancy check (CRC) algorithm. Each integer corresponds to a bin in an integer array of fixed length L (usually within 12 default hologram lengths of 53, 59, 61, 71, 83, 97, 151, 199, 257, 307, 353, and 401 bins). Thus, all generated fragments are hashed into array bins in the range of 1 to L. This array now constitutes a molecular hologram, the bin occupancies being the descriptor values. The following standard PLS analysis identifies a set of explanatory variables [20,21,22]. 

## 4. Conclusions

By using the holographic QSAR method, four correlation equations with high statistical quality and predictive value could be established. The visualization of the results by contribution maps with special color codings reflects, which combinations of molecular fragments are essentially contributing to the recognition of the NMDA and two 5-HT receptors by Ts. 

## Appendix

**Table A1:** Structures and experimental pIC_50_ values (M), inhibiting the binding of [^3^H]MK-801 in absence and presence of 30 µM spermine, and the binding of [^3^H]ketanserin and of [^3^H]8-OH-DPAT, for a series of Ts. Data are logarithmic transformations of the IC_50_ values published previously [19].

No	Molecular name	pIC_50_			
		[^3^H]MK-801	[^3^H]MK-801 in the presence of 30 µM spermine	[^3^H]ketanserin	[^3^H]8-OH-DPAT
1	tryptamine	4.07	3.32	4.79	6.48
2	homo-T	4.30	3.54	5.12	6.15
3	1-Me-T	4.27	3.37	5.10	6.11
4	2-Me-T	4.96	4.56	4.52	5.81
5	5-Me-T	5.19	5.05	5.00	7.21
6	5-Bu-T	4.82	4.18	5.12	7.15
7	6-Me-T	3.95	3.37	4.34	5.41
8	7-Me-T	4.34	3.56	4.75	5.75
9	α-Me-T	3.62	2.82	4.90	5.80
10	N-Me-T	3.42	2.68	4.91	7.00
11	5-F-T	4.41	3.67	5.21	6.81
12	5-F-hT	4.18	3.34	5.51	6.41
13	5-Cl-T	4.75	3.92	5.17	7.06
14	5-Br-hT	5.13	4.10	5.64	7.49
15	5-OH-T	3.76	3.22	5.27	8.80
16	5-MeO-T	4.72	3.75	4.97	8.23
17	5-NH_2_CO-T	3.66	3.24	3.80	9.82
18	1,2-Me_2_-T	4.79	3.84	4.83	5.84
19	2,5-Me_2_-T	5.43	4.69	4.80	4.77
20	2-Me-5-Et-T	5.81	4.72	5.03	5.28
21	2-Me-5-Bu-T	4.91	4.05	5.08	-
22	2-Me-5-iBu-T	5.22	4.18	5.20	5.60
23	2-Me-5CN-T	4.66	3.53	3.76	5.40
24	2-Me-5-F-T	4.95	4.25	4.66	5.36
25	2-Me-5-Cl-T	5.45	4.41	5.24	5.11
26	2-Me-5-Br-T	5.60	4.65	5.35	5.75
27	2-Me-5-I-T	5.64	4.86	5.27	6.26
28	2-Me-5-OH-T	4.27	3.35	3.72	5.67
29	2-Me-5-MeO-T	5.28	4.29	4.28	5.95
30	2,7-Me_2_-T	3.81	3.20	4.75	5.28
31	2-Me-7-CF_3_-T	4.05	3.26	5.48	-
32	2-Me-7-Et-T	4.00	3.35	4.74	-
33	2-Me-7-iPrp-T	4.39	3.64	4.69	5.39
34	2-Me-7-F-T	4.74	4.05	4.83	-
35	2-Me-7-Cl-T	4.38	3.75	5.54	5.69
36	2-Me-7-Br-T	4.21	3.45	5.51	-
37	2-Me-7-I-T	4.43	3.75	5.52	-
38	5-Me-7-Br-T	4.68	4.00	5.37	6.01
39	α -Me-5-F-T	4.19	3.31	5.15	5.90
40	α -Me-5-OH-T	3.17	2.39	5.09	7.41
41	4,6-Cl_2_-T	4.32	3.83	5.26	5.74
42	2,4,6-Me_3_-T	3.61	3.31	4.28	-
43	2,4,7-Me_3_-T	3.74	3.09	5.38	5.54
44	2,5,7-Me_3_-T	4.20	3.29	4.74	4.84
45	2,6,7-Me_3_-T	4.15	3.30	4.75	5.07
46	2,5-Me_2–7_-Br-T	4.41	3.68	5.47	-
47	2,7-Me_2–4_-F-T	5.26	4.54	5.48	6.00
48	2,7-Me_2–4_-Cl-T	3.90	3.45	5.69	6.29
49	2,7-Me_2–5_-Cl-T	4.52	3.75	5.12	-
50	2,7-Me_2–6_-Cl-T	4.33	3.55	4.90	-
51	2-Me-5-Br-7-Et-T	4.43	3.88	5.03	-
52	2-Me-5,7-F_2_-T	4.84	3.99	5.24	-
53	2-Me-4,7-Cl_2_-T	4.41	3.88	5.92	6.37
54	2-Me-5,7-Cl_2_-T	4.63	3.94	5.28	-
55	2-Me-6,7-Cl_2_-T	4.60	3.88	5.43	-
56	2-Me-4,7-Br_2_-T	4.47	4.06	5.55	6.76
57	2-Me-5-F-7-Br-T	4.41	3.64	5.35	-
58	2-Me-5-Br-7-F-T	5.57	4.81	5.24	5.56
59	2-Me-5-Cl-7-Br-T	4.94	4.16	5.14	-
60	2-Me-5,7-Br_2_-T	4.87	4.18	5.39	-
61	L-Trp-Me	3.86	2.90	4.40	4.80
62	D-Trp-Me	3.24	2.37	4.44	3.96
63	L-Trp-NH_2_	4.08	3.05	2.96	3.08
64	Trp-Oct	4.71	4.23	5.05	4.36

T, tryptamine; hT, homotryptamine [3-(1*H*-indol-3-yl)propan-1-amine]; - , not measured.
